# The Defective and Dependent

**Published:** 1912-08-24

**Authors:** 


### THE DEFECTIVE AND DEPENDENT.*

### XII.—Relations of Mental-Defectiveness to Unemployment, Immorality, and Crime.

The urgency in the public interest of such legislation as was referred to in our last article may be illustrated by a consideration of the social evils which result from the present imperfect control of such mental-defectives as are not within the purview of the existing laws dealing with lunatics and idiots.

### Unemployment.

We have seen (The Hospital, May 4, 1912, p. 132) that in the districts in which the Act of 1899 has been adopted special training has been serviceable in fitting a varying percentage of the feeble-minded pupils of the elementary school class to follow simple industrial pursuits after leaving school at sixteen years of age; but after-care investigations tend to show that even in improved cases there is risk of unemployment resulting from the fact that inherent lack of initiative and self-directing power renders them unfit to maintain for any length of time competition with their normal fellows in the open labour market. Some, indeed, never get beyond the "blind-alley" occupations of errand-boys and "van-guards," upon which they started on leaving school; and, if employed as labourers or in simple handicrafts, they are the first to be discharged when work becomes slack and the last to regain their jobs. Girls who have been trained for domestic service in Feeble-Minded Homes are seldom a permanent success, as their disabilities and peculiarities of disposition are apt to create friction in households, so that their retention, even by considerate mistresses, becomes difficult. These poor girls consequently pass from pillar to post, and in the absence of adequate protection end their career on the streets.

### Immorality.

In both sexes, indeed, the descent from unemployment to immorality and crime is but too easy. Apart from innate vicious tendencies which exist in a certain number of the feeble-minded, the feebleness of will and the facility to suggestion which characterise the class render them more likely than others to fall into vicious courses. The Medical Investigators employed by the Royal Commission and Local Government Board Inspectors who gave evidence concur in testimony as to the frequency with which feeble-minded women are found in the maternity wards of workhouses.

Mr. Bagenal, Inspector for the Yorkshire District, states: "In one workhouse I found five young women, all of whom were feeble-minded. Number one was going to be confined, and had had two children before; number two had had two children; number three had had two children; number four had had one child; and number five had been confined in the summer, and had three children previously. All these were illegitimate. The cost of these cases is a very great burden to the ratepayers, especially as the children will probably turn out to be feeble-minded also. The fact is this class become practically the prostitutes of the rural districts." In an investigation made some years ago by the National Vigilance Society, it was found that during one year 715 feeble-minded women passed through 105 workhouses (as "ins-and-outs" who could not legally be detained against their will), while at fifty-six workhouses there were 366 such women known to have been leading immoral lives; and Mr. W. A. Coote, the Secretary of that Association, recently stated his belief that "an examination of the inmates of our Rescue Homes by a committee of medical experts would show that a large proportion of this trouble is owing to, or has its origin in, feeble-mindedness, in some form of brain weakness, rather than what we are accustomed to regard as sin, and as the outcome of a corrupt and vicious nature."

### Inebriety.

The evidence given before the Royal Commission led them to place much stress upon the relation of habitual inebriety to mental defect, and to record their opinion that "probably some 60 to 70 per cent. of the habitual inebriates who are dealt with under the Acts are mentally-defective."

Indeed, it would appear from the Report of the Inspector under those Acts, to which reference is made, that, out of 1,873 admissions to inebriate reformatories, not more than 697 could be considered to be of average mental capacity. The hereditary influence of parental alcoholism in the production of mentally-inferior offspring must also be taken into account in this connection; and, although considerable divergence of opinion exists as to how far parental alcoholism *per se* can be justly regarded as a predominant factor in the production of defective children, there can be no doubt that when combined with neuropathic predisposition it may be accounted as responsible for a large percentage of congenital mental defect.

### Crime.

As shown by prison statistics the close connection of mental-defectiveness and crime is strikingly apparent. The Medical Investigators to the Royal Commission showed that about 10 per cent. of those examined by them in various prisons were feeble-minded. Dr. Melland has stated that of 1,048 prisoners seen by him in Strangeways Gaol at Manchester, no less than 139 were, in his opinion, mentally affected, though, as there might be doubt as to original defectiveness in twenty-eight alcoholic inmates, he deducted the latter and put down the

* Previous articles appeared on November 4, 18, December 2, January 6, February 10, March 2, 23, May 4, June 15, July 13, August 10.

percentage as 10 rather than 13. He found that the majority of these were under short sentences for trivial offences, such as petty thefts, drunkenness, begging, or indecency, and in not a few there had been repeated convictions for the same class of offence. He remarks that the majority of these feeble-minded prisoners are the victims not so much of inborn criminal tendencies as of weakness of will rendering them facile and easily impelled to follow the line of least resistance under adverse circumstances.

Limits of space prevent our further pursuing this aspect of the subject, but sufficient evidence has been adduced to show the need of early and effectively continued control to prevent those who are defective by no fault of their own from passing into the ranks of the delinquent. The Mental Deficiency Bill now before Parliament seeks, by timely segregation in suitable institutions and labour colonies of those not under adequate control, to effect this object; and with the necessary safeguards against abuse of the liberty of the subject it may become a beneficent measure, not only in the social economics of the present generation but, by checking the uncontrolled propagation of the unfit, diminish the evil in the future.

